# Approach to the Patient: “Utility of the Copeptin Assay”

**DOI:** 10.1210/clinem/dgac070

**Published:** 2022-02-08

**Authors:** Mirjam Christ-Crain, Julie Refardt, Bettina Winzeler

**Affiliations:** 1 Department of Endocrinology, Diabetes and Metabolism, University Hospital Basel, 4031 Basel, Switzerland; 2 Department of Clinical Research, University Hospital Basel, 4031 Basel, Switzerland

**Keywords:** copeptin, diabetes insipidus, primary polydipsia, SIAD, hyponatremia, hypernatremia, diagnosis

## Abstract

Copeptin derives from the same precursor peptide preprovasopressin as arginine vasopressin (AVP). The secretion of both peptides is stimulated by similar physiological processes, such as osmotic stimulation, hypovolemia, or stress. AVP is difficult to measure due to complex preanalytical requirements and due to technical difficulties. In the last years, copeptin was found to be a stable, sensitive, and simple to measure surrogate marker of AVP release. Different immunoassays exist to measure copeptin. The 2 assays which have most often be used in clinical studies are the original sandwich immunoluminometric assay and its automated immunofluorescent successor. In addition, various enzyme-linked immunosorbent assay have been developed. With the availability of the copeptin assay, the differential diagnosis of diabetes insipidus was recently revisited. The goal for this article is therefore to first review the physiology of copeptin, and second to describe its use as marker for the differential diagnosis of vasopressin-dependent fluid disorders, mainly diabetes insipidus but also hyper- and hyponatremia. Furthermore, we highlight the role of copeptin as prognostic marker in other acute and chronic diseases.

## Case Presentation

A 25-year-old male patient came to our outpatient clinic for evaluation of polyuria/polydipsia syndrome. Over the last weeks, he noticed slowly progressive polydipsia due to increased thirst sensation. At the time of presentation, he was drinking 8 L of water per day and complained of polyuria with emictions every 1 to 2 hours and nocturia 2 to 4 times a night. Several trials of reducing his fluid intake had led to headache and vertigo.

Three months preceding the presentation, the patient was in a scooter accident and suffered a minor head trauma. Since this episode, he has noticed increasing feelings of tiredness and low energy. Besides this accident, no comorbidities were known. There was no medication or drug intake. His grandmother had type 1 diabetes and his grandfather leukemia, otherwise the family history was unremarkable.

Physical examination revealed a normal weight (weight 58 kg, height 170 cm, body mass index 21 kg/m^2^), normotensive (blood pressure 124/83 mmHg), and normocardiac (63 beats per minute) patient. Neurological examination was unremarkable. Eczema was seen on both hands and wrists.

Baseline laboratory showed normonatremia with a plasma sodium level of 140 mmol/L; plasma glucose (4.6 mmol/L), calcium (2.4 mmol/L), and potassium (3.9 mmol/L) were also within the normal range. Hormonal status revealed intact anterior pituitary function.

For further evaluation, he underwent a classical water deprivation test (Table 1). After an overnight fast (food and drinks) from midnight onwards, the patient came to our clinic at 8 am. Laboratory evaluations showed plasma sodium of 141 mmol/L and plasma osmolality of 293 mosm/kg with a low urine osmolality of 118 mosm/kg. Over the next 8 hours, the patient lost around 3% of his body weight with total urinary excretion of 1220 mL. Plasma sodium increased to 143 mmol/L with plasma osmolality of 289 mosm/kg, while urine osmolality increased to 481 mosm/kg. At this time point, the patient was given 2 µg of desmopressin intravenously, after which urine osmolality after 1 hour increased to 650 mosm/kg, corresponding to a 35% increase. Based on these results, the patient was diagnosed with partial central diabetes insipidus and treatment with desmopressin nasal spray 10 µg before bedtime was started. Further evaluation with magnetic resonance imaging of the pituitary showed an intrasellar microadenoma of 5 × 6 mm. The typical bright spot—an area of hyperintensity in the posterior pituitary believed to result from stored arginine vasopressin (AVP)—was not visible. Accordingly, post-traumatic partial central diabetes insipidus was considered the most likely cause.

**Table 1.** Water deprivation test of case patient

	Time					
	8 am	10 am	12 pm	2 pm	4 pm	5 pm
Weight, kg	57.9	57.4	56.8	56.6	56.3	56.3
P-osmolality, mosm/kg	293				289	
P-sodium, mmol/L	141				143	
Urinary excretion, mL	390	290	300	140	70	30
U-osmolality, mosm/kg	118	131	225	443	481	650

Water deprivation test was started after an overnight fast (food and drinks) from midnight onwards. After sampling at 4 pm, the patient received 2 µg of desmopressin intravenously.

Abbreviations: P, plasma; U, urine.

At the 3-month follow-up visit, the patient was happy with the desmopressin treatment, under which his thirst sensation had decreased and he had normalized his fluid intake. Repeated plasma sodium levels were between 140 and 142 mmol/L.

Six months after starting desmopressin treatment, the patient presented as an emergency with severely reduced wellbeing with constant headache, vertigo, tiredness, and nausea. Due to a renewed increase in thirst with subsequent increased fluid intake of up to 7 L per day, he had doubled his desmopressin dose. Laboratory analyses showed hypotonic hyponatremia with plasma sodium of 126 mmol/L and plasma osmolality of 256 mosm/kg. After stopping desmopressin, the symptoms ameliorated quickly and sodium levels normalized. Two weeks later, however, the patient wanted to resume desmopressin treatment because he experienced a severe feeling of thirst with accompanying polyuria and polydipsia. What is the patient’s diagnosis now? Should desmopressin be resumed? Are there additional tests to address these questions? The following mini-review will address these and other questions. The patient’s subsequent course will be described at the end.

## Introduction

Copeptin derives from the same precursor peptide preprovasopressin as AVP. AVP is difficult to measure due to complex preanalytical requirements and for technical reasons (1). Therefore, despite great expectation for AVP to become a clinical routine marker in differentiation of fluid disorders, measurement of AVP has never entered everyday practice. Copeptin was found within the last years to be a stable, sensitive, and simple to measure surrogate marker of AVP release (2, 3).

**Figure 1.**
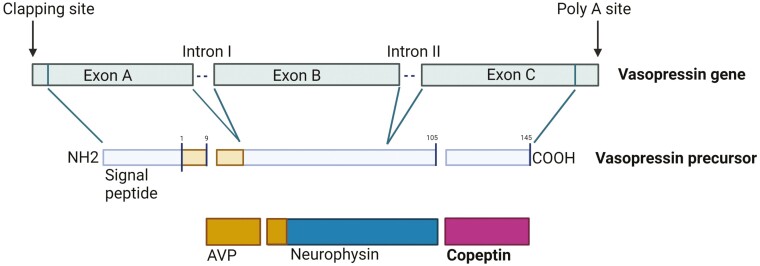
Arginine vasopressin (AVP) gene and its protein products. The 3 exons encode a 145 amino acid prohormone with an amino terminal signal peptide. Exon 1 of the AVP gene encodes the signal peptide, AVP, and the NH_2_ terminal region of neurophysin-2. Exon 2 encodes the central region of neurophysin-2, and exon 3 encodes the COOH terminal region of neurophysin-2 and the glycopeptide. The prohormone is packaged into neurosecretory granules of magnocellular neurons. During axonal transport of the granules from the hypothalamus to the posterior pituitary, enzymatic cleavage of the prohormone generates the final products: AVP, neurophysin and the COOH-terminal glycoprotein copeptin. When afferent stimulation depolarizes the AVP-containing neurons, the 3 end products are released into capillaries of the posterior pituitary in equimolar manner (modified from (7)). Created with BioRender.com.

The 164 amino acid precursor protein consists of a signal peptide, the AVP moiety, a protein called neurophysin-2, and a 39 amino acid long glycosylated peptide with a leucine-rich core segment, termed copeptin (Fig. 1) (4, 5). The gene that encodes this precursor in humans is composed of 3 exons and 2 introns located on the short arm of chromosome 20 (20p13) (4, 6). Two different neuroendocrine mechanisms are involved in the production and release of the pro-AVP precursor. In the first, pro-AVP is synthesized in the magnocellular neurons, and processed in the endoplasmic reticulum by the removal of the signal peptide and the addition of a carbohydrate chain. Additional post-translational processing occurs during the transport of the precursor protein down to the axon terminals in the posterior pituitary where the peptides are enzymatically cleaved and stored in the posterior pituitary.

A second AVP neurosecretory pathway transports high concentrations of the hormone from parvocellular neurons to the pituitary portal system where AVP acts synergistically with corticotropin-releasing hormone to stimulate adrenocorticotropic hormone release from the anterior pituitary (8, 9). This “backup” system of 2 hormones from the hypothalamus for the release of adrenocorticotropic hormone underlines the physiological importance of the endocrine stress response. It also defines AVP and copeptin as potential biomarkers to measure the level of stress and disease severity and consequently to act as a prognostic marker in different diseases (see below).

Both peptides are stimulated by similar physiological processes, such as osmotic stimulation, hypovolemia, or stress. However, while the physiological function of AVP is homeostasis of fluid balance, vascular tonus, and regulation of the endocrine stress response, the physiological function of copeptin is much less clear. Early experiments debated the role of copeptin as a prolactin-releasing factor with inconclusive results (10, 11). More recent data postulate copeptin to be a chaperone-like molecule which is involved in the structural formation of pro-AVP (12). Copeptin is reported to interact with the calnexin/calreticulin system (13, 14), which monitors protein folding and interacts with glycosylated proteins. This decreases the formation of inactive and increases the formation of active hormones. It is tempting to attribute the inefficient monomer folding in the absence of copeptin to the pathogenesis of central diabetes insipidus, but this needs further examination. The tight regulation of copeptin in the circulation raises the idea that copeptin may have a specific peripheral function, although experimental data show no evidence for this. Importantly, copeptin responds as rapidly as AVP to osmotic, hemodynamic, and unspecific stress-related stimuli which may be explained by its equimolar production together with AVP.

Thus, providing that an AVP assay of high quality is used, copeptin shows a strong correlation with AVP. Using a well-established AVP assay (2), a direct comparison between copeptin and AVP release in relationship to serum osmolality showed a very strong correlation between both peptides (r = 0.8) and a stronger correlation for serum osmolality with copeptin (r = 0.77) than with AVP (r = 0.49). Analyses with a commercial AVP radioimmunoassay show a much lower correlation from the equimolar release of both peptides (r = 0.31) (3).

Studies on elimination kinetics are scarce. One recent study suggested a 2 times longer half-life for copeptin in relation to AVP (15). These different half-lives most probably reflect the metabolic clearance rates of these peptides. In contrast to AVP which is inactivated by plasma and tissue endopeptidases in the kidney and liver, the catabolism of copeptin has never been investigated. The fact that copeptin does not accumulate as a junk protein in the circulation, and that its elimination stops once removed from the circulation argues against the role of circulating proteases. Data show that copeptin is at least partially eliminated by the kidneys (16). Accordingly, in patients with chronic kidney disease, copeptin levels inversely correlate with decreasing glomerular filtration rate, suggesting renal clearance of copeptin by the kidneys (17).

In normo-osmotic baseline conditions, median copeptin plasma concentrations were shown in healthy volunteers to be 4.2 pmol/L (median) with a broad range between 1 and 13.8 pmol/L (3, 18). Men show slightly higher values than women, but with the difference in median values of only about 1 pmol/L (3, 18). Copeptin levels show no correlation with age (3), and no circadian variability (19). Copeptin release seems not to be affected by food intake (20) or the menstrual cycle of women (21), suggesting that copeptin measurements are quite robust and can be reliably interpreted independently of time point of withdrawal, prandial status, or menstrual cycle.

**Figure 2.**
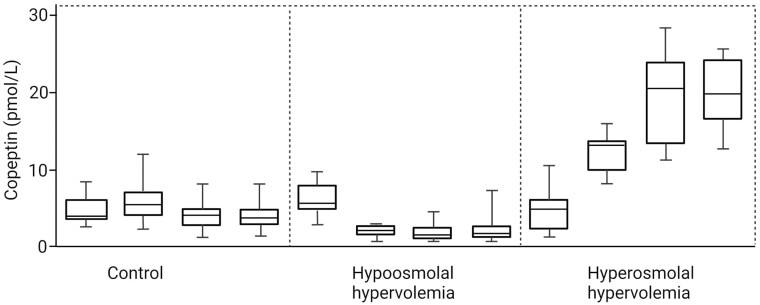
Copeptin levels in hypo-osmolal hypervolemia: the study subjects received 4 µg of desmopressin intravenously at 20:00 hours and at 08:00 hours on the following day. Simultaneously, they were instructed to drink 2 to 2.5 L of tap water during the night. Copeptin levels in hyperosmolal isovolemia: study subjects received 1 mL/kg/hour of 2% saline infusion from 20:00 hours to 08:00 hours, and 200 mL/hour of 5% saline infusion from 08:00 hours to 13:00 hours. They were instructed not to drink during the whole study period. The control experiment consisted of access to oral water ad libitum during the whole study period (modified from (22)). Boxplots indicate median and IQR and whiskers represent the range. Created with BioRender.com.

Of note, however, small amounts of oral fluid intake significantly decrease copeptin levels, which is important to notice for data interpretation (20). As expected, copeptin shows the same behavior to osmotic and hemodynamic stimuli as demonstrated for AVP. Water deprivation alone and followed by a hypertonic saline infusion increases plasma copeptin, whereas decreasing serum osmolality through hypotonic saline infusion or an oral water load decreases plasma copeptin, respectively (2, 22) (Fig. 2).

## Measurement of Copeptin

Different immunoassays exist to measure copeptin. The 2 assays which have most often be used in clinical studies are the original sandwich immunoluminometric assay (LIA) (3) and its automated immunofluorescent successor (on the KRYPTOR platform). In addition, various enzyme-linked immunosorbent assays (ELISAs) have been developed (23, 24). A recent study showed that copeptin measured by the KRYPTOR and LIA assays showed a good correlation over a wide range of copeptin concentrations (from very low to very high levels). In contrast, copeptin measured by the ELISA correlated only poorly with both the KRYPTOR and LIA measured copeptin concentrations. Importantly, for the current main indication of copeptin measurement (ie, differential diagnosis of polyuria polydipsia syndrome, see below) the cut-offs for copeptin have been developed and validated with the LIA and KRYPTOR assay; the ELISA has a poor diagnostic accuracy using these cut-offs, with an especially low sensitivity in correctly diagnosing patients with central diabetes insipidus (25). Therefore, for this indication, and also for other indications where cut-offs have been developed with KRYPTOR or LIA, further studies with development and validation of cut-offs are needed if the ELISA is used.

## Utility of Copeptin Measurement in the Differential Diagnosis of Diabetes Insipidus

### Differential Diagnoses of Polyuria–Polydipsia Syndrome

As discussed above, AVP is the main regulatory hormone for salt-water homeostasis. Stimulated by an increase in plasma osmolality or hypovolemia, AVP induces water reabsorption via V2 receptors in the kidneys (26). Impairment of this process leads to diabetes insipidus, characterized by polyuria of more than 50 mL per kg body weight per 24 hours of hypotonic urine and polydipsia of more than 3 L a day (26). Diabetes insipidus is further divided into a central and a nephrogenic origin. While the first cause is due to impaired AVP production or secretion upon osmotic or nonosmotic stimulation (27, 28), the second cause results from renal resistance to AVP (29).

Central diabetes insipidus is mainly caused by destruction of the producing or secreting cells, leading to a partial or complete form. The main causes are accordingly pituitary surgery, head trauma, and infiltrative diseases such as sarcoidosis or metastases (30, 31); inflammatory or autoimmune disorders such as hypophysitis (32) and familial forms due to a genetic defect leading to impaired AVP synthesis (33, 34) are also known. A rare form, gestational diabetes insipidus, manifests during pregnancy and is caused by increased AVP metabolism due to the placental enzyme vasopressinase (35, 36).

Nephrogenic diabetes insipidus is mainly secondary to adverse drug effects (lithium), electrolyte disturbances (hypercalemia or hypokalemia) or kidney diseases (29), resulting in renal insensitivity to AVP and corresponding lack of aquaporin 2–mediated water reabsorption. If discovered early on, these secondary forms are often reversible in contrast to rare congenital forms due to mutations in the *AVPR2* or the *AQP2* genes (37).

An important differential diagnosis to central or nephrogenic diabetes insipidus is primary polydipsia. Here, polyuria–polydipsia occurs despite intact AVP secretion and renal sensitivity. As a result of this chronic excessive fluid intake, the concentration gradient in the renal medulla is continuously reduced with a downregulation of aquaporin 2 channels (38). Chronic central AVP suppression may also play a role. These factors lead to the excretion of hypotonic urine, making it difficult to distinguish it from diabetes insipidus. Primary polydipsia has been increasingly observed in health conscious people who aim for a high daily water intake; other possible triggers are dependency disorders or a reduced thirst threshold. Severe polydipsia has primarily been described in psychiatric patients (39, 40).

### Copeptin Based Tests to Differentiate Diabetes Insipidus From Primary Polydipsia

Given the different etiologies of these disorders, there are major differences in treatment. For example, while central diabetes insipidus is mainly treated with exogenous AVP (desmopressin), patients with primary polydipsia are advised to slowly reduce their fluid intake. Therefore, a diagnostic test with high diagnostic accuracy is crucial to avoid wrong treatment decisions. While the standard water deprivation test as described for our patient has been the diagnostic gold standard for decades despite its low diagnostic accuracy of only 70% (28), recent evaluations showed improved diagnostic accuracy for copeptin based tests.

The easiest etiology to diagnose is nephrogenic diabetes insipidus, since copeptin measurements of these patients revealed consistently high levels. In fact, it has been shown that using an unstimulated copeptin cut-off of >21.4 pmol/L had a 100% sensitivity and specificity to diagnose nephrogenic diabetes insipidus (41). Accordingly, no further evaluations than a random copeptin measurement are needed in these patients.

Unfortunately, the distinction between central diabetes insipidus and primary polydipsia is not quite so straightforward because of the considerable overlap in baseline copeptin levels. In particular, distinguishing partial central diabetes insipidus from primary polydipsia is very challenging (26, 42). Two new copeptin-based test procedures with high diagnostic accuracy have been proposed to overcome these difficulties: the hypertonic saline stimulation test (43) and the arginine stimulation test (44).

Using osmotically stimulated copeptin levels as a diagnostic measure for diabetes insipidus was first described by our group in a study involving 55 patients with polyuria–polydipsia syndrome (41). Here, the cut-off value for copeptin of 4.9 mmol/L, measured after a rise in plasma sodium levels above 147 mmol/L, showed high diagnostic accuracy in distinguishing central diabetes insipidus from primary polydipsia (41). We have recently validated this derived copeptin cut-off level in an international multicenter study including 156 patients with polyuria–polydipsia syndrome (43). For this study, plasma sodium levels were increased up to 150 mmol/L by an infusion of 3% hypertonic saline adjusted for body weight. The diagnostic value of the osmotically stimulated copeptin level was confirmed, with a threshold of 4.9 pmol/L showing a diagnostic accuracy of 97% for the diagnosis of central diabetes insipidus (Table 2). The diagnostic accuracy was only slightly lower with 95% when comparing partial central diabetes insipidus with primary polydipsia (43). In addition, the test proved to be safe and well tolerated, with a majority of patients preferring it over the standard water deprivation test (43).

**Table 2.** Overview copeptin cut-offs

Test/Indication	Time point measurement	Copeptin cut-off	Test performance	Reference
Random copeptin measurement	Any time	>21.4 pmol/L = nephrogenic diabetes insipidus	Diagnostic accuracy 100%	Timper K, et al. *J Clin Endocrinol Metab* 2015 (41)
Hypertonic saline infusion test	Once plasma sodium ≥147 mmol/L	≤4.9 pmol/L = central diabetes insipidus	Diagnostic accuracy 97%	Fenske W, et al. *N Engl J Med* 2018 (43)
		>4.9 pmol/L = primary polydipsia		Timper K, et al. *J Clin Endocrinol Metab* 2015 (41)
Arginine infusion test	60 minutes after start of arginine infusion	≤3.8 pmol/L = central diabetes insipidus	Diagnostic accuracy 93%	Winzeler B, et al. *Lancet* 2019 (44)
		>3.8 pmol/L = primary polydipsia		
Pituitary surgery stimulated copeptin	1st postoperative day	<2.5 pmol/L = central diabetes insipidus	Positive predictive value 81%, specificity 97%	Winzeler B, et al. *J Clin Endocrinol Metab* 2015 (48)
		>30 pmol/L = no central diabetes insipidus	Negative predictive value 95%, sensitivity 94%	
Hypernatremia in hospitalized patients	Plasma sodium >155 mmol/L	≤4.4 pmol/L = central diabetes insipidus	Sensitivity 100%, specificity 99%	Nigro N, et al. *Crit Care* 2018 (49)
Hyponatremia in hospitalized patients	Plasma sodium <125 mmol/L	>84 pmol/L = hypovolemic hyponatremia	Sensitivity 23%, specificity 90%	Nigro N, et al. *Clin Endocrinol* 2017 (50)
		<3.9 pmol/L = primary polydipsia	Sensitivity 58%, specificity 91%	

In 2019, a similar study from our group showed that copeptin can also be effectively stimulated with an arginine infusion (44), a test used to stimulate anterior pituitary hormones and in particular to diagnose growth hormone deficiency (45-47). In this study, a copeptin level of 3.8 pmol/L taken 60 minutes after the start of the arginine infusion had a diagnostic accuracy of 93% to distinguish central diabetes insipidus from primary polydipsia (44) (Table 2). Although arginine infusion seems to be a slightly weaker (nonosmotic) stimulus of copeptin and therefore results in somewhat lower diagnostic accuracy, it has several advantages over the hypertonic saline infusion test. It does not require constant rapid monitoring of sodium levels and is, therefore, more practical in clinical routine. Also, the test has a very convenient tolerability profile with mild transient nausea being the only common adverse symptom. More severe symptoms such as vertigo or headache that are often observed during hypertonic saline infusion are rare. However, if severe nausea or vomiting occurs, test results must be evaluated with caution: Since these are potent stimulators of AVP, the occurrence of these symptoms could lead to false-positive results. Therefore, a confirmatory test with hypertonic saline is recommended (44). The pathway by which arginine leads to the release of AVP/copeptin is not entirely clear at present, but a possible activation via the l-arginine–nitric oxide pathway has been suggested (44).

A direct comparison of the 2 tests in a retrospective analysis confirmed the better tolerability, but slightly lower diagnostic accuracy, of the arginine infusion test (44). Currently, we are performing a randomized, multicenter trial comparing the diagnostic accuracy of arginine-stimulated copeptin levels and hypertonic saline–stimulated copeptin levels (NCT03572166) to prospectively investigate this question. First results are expected in 2022.

### Copeptin as a Predictive Marker for Central Diabetes Insipidus

In addition to diagnosing diabetes insipidus in the outpatient clinic, copeptin measurement has also been proposed in the evaluation of postoperative central diabetes insipidus. While 1 study showed a high diagnostic accuracy of 100% for insulin-induced hypoglycemia-stimulated copeptin with a cut-off value of 4.75 pmol/L to detect central diabetes insipidus 3 months after pituitary surgery, the risks of this test outweigh its potential value (51). Other studies used surgery itself as a stress test. In a study including 205 patients of which 49 had a postoperative diabetes insipidus, a copeptin level <2.5 pmol/L measured on the first postoperative day had a positive predictive level of 81% and specificity of 97%. Meanwhile, a copeptin level >30 pmol/L had a negative predictive value of 95% and sensitivity of 94% (48). A second study examined the use of copeptin levels 1 hour after extubation after pituitary surgery. While a cut-off value below 4.2 pmol/L indicated permanent central diabetes insipidus, a value above 12.8 pmol/L was predictive of an unremarkable postoperative course (52). Since only 8 of the included 66 patients had central diabetes insipidus, however, these results should be confirmed in a larger cohort.

In conclusion, copeptin-based tests are valuable diagnostic measures in the difficult distinction between diabetes insipidus and primary polydipsia.

## Utility of Copeptin Measurement in Hyper- and Hyponatremia

### Copeptin Measurement in Hypernatremia

While hypernatremia is a rare finding in outpatients, it is much more common in hospitalized patients showing a prevalence of 2% to 3% (53, 54). Hypernatremia results from a free water deficit due to renal (central or nephrogenic diabetes insipidus, glycosuria) or extrarenal water loss or from iatrogenic sodium overload (eg, uncontrolled saline infusion, tube feeding). In the majority of hospitalized hypernatremia patients, the electrolyte imbalance is not present at admission, but develops during hospitalization (49, 55). Besides iatrogenic issues, many other factors and comorbidities such as heart failure, chronic kidney disease, sepsis, neurological impairment with impaired thirst perception, or inability to drink may promote the development of hypernatremia in the acute setting. Hypernatremia is therefore also a marker of disease severity and, unsurprisingly, studies consistently report increased mortality rates in patients with hypernatremia (56-58).

The most important measure to correct hypernatremia is to increase the free water supply, such as by intravenous hypotonic infusion. Additionally, in salt-overloaded patients iatrogenic action has to be stopped and diabetes insipidus patients need timely treatment with desmopressin.

The high rate of hospital-acquired hypernatremia underpins insufficient hypernatremia awareness in hospitalized patients, with the result of inadequate treatment and worse outcome (57, 59-61). Specifically, for hospitalized patients with diabetes insipidus, a retrospective audit showed that desmopressin treatment was delayed in 88% of patients at admission, putting them at risk of hypernatremia (62, 63). This prompted the Society for Endocrinology to publish guidelines on in-hospital management of central diabetes insipidus in 2018 (64). For patients with undiagnosed diabetes insipidus who are also at need for early desmopressin treatment, copeptin has been proposed as a readily available diagnostic marker in the acute setting.

**Figure 3.**
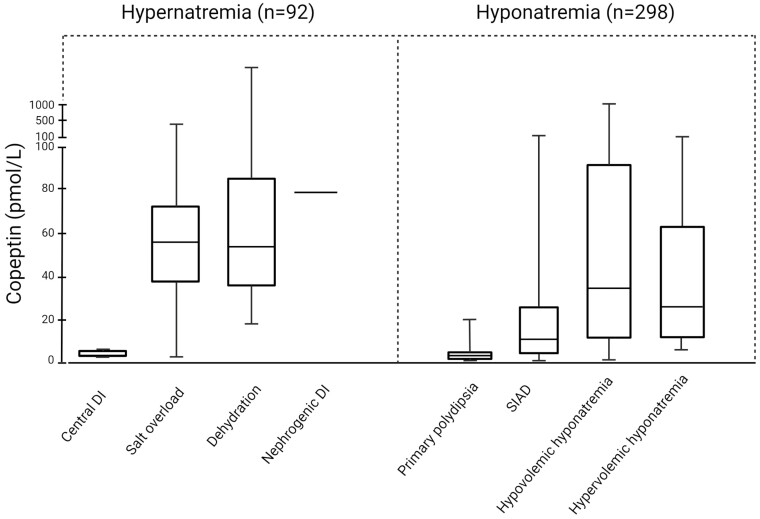
Copeptin levels in the differential diagnosis of hyper- and hyponatremia (modified from (49, 50)). Boxplots indicate median and IQR and whiskers represent the range. DI, diabetes insipidus; SIAD, syndrome of inadequate antidiuresis. Created with BioRender.com.

In a prospective, multicenter, observational study including 92 hospitalized patients with severe hypernatremia (>155 mmol/L), copeptin reliably identified 5 patients with central diabetes insipidus (49). Copeptin values were very low in this group and a cut-off value of ≤4.4 pmol/L showed very high sensitivity and specificity (100% and 99% respectively). In patients with dehydration (n = 65), salt overload (n = 20), and nephrogenic diabetes insipidus, (n = 2) copeptin values were high. The highest values were observed in nephrogenic diabetes insipidus patients (up to 78 pmol/L), but no discrimination between these 3 groups was possible (median [IQR] copeptin values in dehydration and salt overload groups were 55 [35-72] and 53 [34-86], respectively) (49) (Fig. 3). Other commonly used parameters in the differential diagnosis of hypernatremia are volume status and urine osmolality. For the distinction of the hypernatremia categories dehydration vs salt overload the volume status performed well (area under curve 0.88; 95% CI 0.97-1.00). However, no further differentiation between hypovolemic patients with dehydration or diabetes insipidus was possible. Urine osmolality was a good marker to differentiate between (both central and nephrogenic) diabetes insipidus (inadequate low levels: median [IQR] 284 mosm/kg [209-306]) and dehydration or salt overload (adequate high levels: median [IQR] 546 mosm/kg [463-647], and 510 mosm/L [462-819]. The utility of urine osmolality was, however, limited by the fact that urine samples were collected only in 41% of patients (49). In summary, copeptin, together with urine osmolality, is a reliable measure in hypernatremia patients and identifies patients with central diabetes insipidus in need of prompt desmopressin treatment with high diagnostic accuracy.

### Copeptin Measurement in Hyponatremia

Copeptin has also been proposed as a diagnostic marker in hyponatremia (50, 65, 66), which makes sense from a pathophysiological point of view: underlying conditions leading to hyponatremia are assumed to be AVP dependent or AVP modulating and can be classified according to the patient’s volume status (hypervolemic, hypovolemic, and euvolemic hyponatremia). Hypervolemic (due to heart failure, liver cirrhosis, nephrotic syndrome) and hypovolemic hyponatremia (eg, hemorrhage, gastrointestinal losses) are both characterized by decreased effective arterial blood volume, whereas euvolemic hyponatremia with SIAD (syndrome of inappropriate antidiuresis) as the most frequent cause (67, 68) is not.

The diagnostic approach to patients with hyponatremia is unsatisfactory and complex and accurate diagnostic markers would be of major clinical importance since hyponatremia is common (69, 70) and associated with increased morbidity, mortality, and length of hospital stay (71-73). The first prospective study investigating the role of copeptin in the differentiation of hyponatremia included 106 hyponatremic patients and showed no diagnostic utility of copeptin alone, but the ratio of copeptin to urinary sodium helped to discriminate between SIAD and conditions with decreased effective arterial blood volume and, therefore, hemodynamically stimulated AVP release (sensitivity 85%, specificity 87%) (66).

A larger prospective observational study with copeptin measurements in 298 hypo-osmolar hyponatremic patients showed that clearly elevated copeptin levels >84 pmol/L were indicative for hypovolemic hyponatremia (specificity 90%, sensitivity 23%). Conversely, very low levels <3.9 pmol/L were diagnostic for primary polydipsia (specificity 91%, sensitivity, 58%) (50). Although specificity for highest and lowest copeptin values was quite high, sensitivity was poor as most patients harbored copeptin measurements within the middle range. Those values overlapped widely between the different hyponatremia categories and copeptin was not helpful in differentiating between SIAD and other main causes of hyponatremia, such as hypovolemic and hypervolemic or diuretic-induced hyponatremia. Moreover, copeptin values also showed a broad variability within single categories (Fig. 3).

### Copeptin Measurement in SIAD

SIAD is characterized by water retention and secondary natriuresis (74) and may arise from conditions such as infections, central nervous system disorders, drugs, pain, or stress and an important number also by cancers (75). The original term “syndrome of inappropriate antidiuretic hormone secretion (SIADH)” introduced by Schwartz et al (76) in 1957 implies that this syndrome is a consequence of elevated AVP release. However, in 1980 4 different types of SIAD were first described according to different osmoregulatory defects (77), but not all of them were associated with elevated AVP levels. The first type was coupled with autonomous, erratic hypersecretion of AVP, while the second type was characterized by a preserved linear correlation between AVP and serum osmolality, but a low osmotic threshold (reset osmostat). The third and fourth types were linked to a constant nonsuppressible AVP leak and hypovasopressinemic antidiuresis, respectively.

In 2014 Fenske et al analyzed serial measurement of copeptin during a hypertonic saline infusion test in SIAD patients (78) and confirmed the 4 subtypes. Moreover, a fifth type with linear decrease in copeptin concentrations with increasing serum osmolality (“barostat reset”) was described.

While in the first study no association of the osmoregulatory defects and clinical signs and symptoms were observed, the second study revealed that the first type (“subtype A”) was primarily found in patients with lung cancer. These patients showed persistently high copeptin values (>38 pmol/L) suggestive of autonomous extra-hypothalamic paraneoplastic production of AVP (eg, in lung cancer tissue) (79-81). This led to the hypothesis that copeptin could be a marker to detect cancer-related SIAD, which is the case in up to one-third of SIAD patients according to the Hyponatremia Registry (82).

In a retrospective study of 146 hospitalized SIAD patients, special attention was paid to patients with cancer-related SIAD (n = 39). Those patients were predominantly male and of younger age and had significantly higher serum sodium and urine osmolality levels than patients with other types of SIAD. Although patients with small cell lung cancer tended to have the highest copeptin values (up to 380 pmol/L) compared with other cancer entities, copeptin values were not consistently elevated in this cancer subgroup nor in cancer-related SIAD in general (83). One explanation of the vast overlap of copeptin levels may relate to the fact that cancer patients have many other reasons for enhanced AVP secretion: comorbidities, medication, or symptoms such as vomiting, nausea, dehydration, or stress (84-87). The unspecific nonosmotic stress–related copeptin stimulus in acute hospitalized hyponatremic patients may, therefore, overrule the osmotic or paraneoplastic impulse (88, 89).

### Nephrogenic Syndrome of Inappropriate Antidiuresis

As a final point, the fourth osmoregulatory SIAD subtype (“subtype D”) (77, 78) with undetectable or low AVP/copeptin values had been attributed to the rare nephrogenic syndrome of inappropriate antidiuresis (90). This syndrome is caused by x-linked recessive gain of function variants in the *AVPR2* gene, encoding the vasopressin V2 receptor (91) or potentially by a pathogenic variant in the *GNAS* gene (92). A consequence of the continuous activation of the vasopressin receptor leading to AVP-independent antidiureses, AVP/copeptin levels are low or suppressed in these patients (92-94).

In summary, copeptin is not a helpful marker in the differential diagnosis of hyponatremia, nor useful to discriminate between different SIAD subtypes including cancer-related SIAD.

## Utility of Copeptin Measurement in Other Diseases

As mentioned above, besides osmotic stimulation of AVP, a second neurosecretory pathway transports high concentrations of AVP from parvocellular neurons to the pituitary portal system, where it acts synergistically with corticotropin-releasing hormone to stimulate release of adrenocorticotropic hormone from the anterior pituitary (8, 9). Therefore, somatic stress nonspecifically increases copeptin concentrations. Indeed, in a study looking at copeptin and cortisol concentrations in 3 groups with increasing stress levels (ie, healthy volunteers without stress, hospitalized medical patients with moderate stress, and surgical patients immediately after extubation with maximal stress), copeptin levels showed a step-wise increase, correlating with cortisol concentrations (95).This positive correlation of copeptin with the individual stress level, mirroring disease severity, builds the rationale for a presumed prognostic value of copeptin in several acute diseases. For example, copeptin has been shown to be a prognostic biomarker in sepsis (96), pulmonary (97, 98), cardiovascular (99, 100), neurological (86, 101), hepatic (102), and urinary (103) diseases.

Copeptin has been shown to gradually increase in different stages of sepsis (ie, systemic inflammatory response syndrome, sepsis, severe sepsis, septic shock) (104) reaching levels as high as 40 times higher than the values of healthy controls (104). Several studies suggest a potential prognostic role of copeptin in sepsis by showing higher levels in nonsurvivors than survivors of sepsis (105, 106). Also, a study in patients admitted to the emergency department showed that copeptin was an independent predictor for development of septic shock (106).

In lower respiratory tract infections, especially community-acquired pneumonia, copeptin levels were shown to be significantly higher than in healthy controls with highest levels in patients with pneumonia (107). During the last years, copeptin has repeatedly been shown to accurately predict mortality independently of clinical risk prediction by various scores (for references see (108):). The largest study including more than 1000 patients showed a superior prognostic accuracy of copeptin for 28-day mortality than the commonly used clinical scores as well as the inflammatory biomarkers C-reactive protein and procalcitonin. Similarly, in patients with acute exacerbations of chronic obstructive pulmonary disease, copeptin concentrations were shown to be predictive for long-term clinical failure independent of age, comorbidity, hypoxemia, and lung functional impairment in multivariate analysis (109).

In patients with heart failure, higher copeptin levels point to a more unfavorable outcome and increased mortality (110-112). The combination of copeptin with brain natriuretic peptide, the standard biomarker for the diagnosis of heart failure, was able to further improve outcome prediction (110).

In patients with acute myocardial infarction (113), copeptin levels were shown to be higher in patients who died or were re-admitted with heart failure compared with survivors. A dual-marker strategy using troponin T, a marker of cardiac necrosis, and copeptin, for rapid rule out of myocardial infarction showed that the combination of troponin T and copeptin resulted in a high diagnostic accuracy in the diagnosis of acute myocardial infarction at presentation to the emergency department. However, the utility of the dual-marker strategy is now questionable with the availability of the high-sensitive troponin assay (114).

In stroke, copeptin emerged as a strong and independent prognostic marker for functional outcome and death, with a superior accuracy than commonly measured laboratory parameters, such as blood glucose, C-reactive protein, and white blood cell count, as well as clinical measures (86). A biomarker-based clinical score including copeptin was externally validated and calibrated showing a better prognostic accuracy than the same score without copeptin (115).

In autosomal dominant polycystic kidney disease (ADPKD) a marker predicting outcome and allowing early interventions would also be urgently needed since this disease ultimately leads to the development of end-stage renal disease in most patients by irreversible loss of glomeruli (116). The natural course of ADPKD varies considerably, within and between affected families. AVP leads to production of cyclic adenosine monophosphate, which is known as a stimulator of cyst formation and growth. A large cross-sectional study in patients with ADPKD showed that copeptin levels correlated with glomerular filtration rate and renal blood flow, independent of age, sex, and diuretic use (117). In another study, copeptin predicted a decrease in glomerular filtration rate during long-term follow-up (118). Recently, a study suggested that measurement of copeptin in subjects with ADPKD could not only help select those at higher risk of rapid progression but also those expected to benefit the most from treatment with vaptans (119).

Finally, copeptin levels in patients with type 2 diabetes showed a positive association of copeptin with blood glucose (120). In addition, high copeptin levels in patients with diabetes predicted a decline in kidney function during follow-up (116). Also, copeptin measured in patients with newly diagnosed type 2 diabetes was a predictor for development of chronic kidney disease during a 10-year follow-up (121). Copeptin was also associated with the risk of severe renal outcomes independent of relevant covariates such as age, duration of diabetes, blood pressure, and baseline levels of HbA1c (122). In patients with type 1 diabetes, a recent study showed that higher copeptin levels independently predicted a decline in renal function.

Different pathophysiological mechanism are discussed; 1 possible explanation could be a blunted response to AVP in the collecting ducts due to a lower abundance of the V2 receptors through which AVP exerts its effects on water reabsorption (123, 124).

## Back to Patient Presentation

Although the water deprivation test initially indicated partial diabetes insipidus, the further course of the disease under desmopressin treatment was suspicious for primary polydipsia. Therefore, our patient underwent the hypertonic saline infusion test for further evaluation. During osmotic stimulation to a plasma sodium level of 150 mmol/L, his copeptin level rose to 16 pmol/L. Accordingly, the diagnosis of diabetes insipidus was revised to primary polydipsia and the patient referred to our colleagues from the psychosomatic department. With their help, the patient was able to slowly decrease his daily fluid intake to 3 L per day. During the last follow-up, the sodium levels remained stable and the patient reported wellbeing. Occasionally, he felt an increased sense of thirst, but he learned to deal with it.

This case illustrates that clinical signs and symptoms, such as severe polyuria–polydipsia with a high daily fluid intake or nocturia that are sometimes described as characteristic of diabetes insipidus, are unspecific (43). Similarly, the magnetic resonance imaging finding “absence of posterior pituitary bright spot” is not specific for central diabetes insipidus as the bright spot is also invisible in up to 40% of primary polydipsia patients (compared to 70% of patients with central diabetes insipidus) (43).

Several observations during the water deprivation test wrongly led to the diagnosis of partial diabetes insipidus: high amount of polyuria and loss of body weight, moderate increase of urine osmolality during test period and good urine osmolality response to desmopressin. At this point it must be mentioned that the diagnostic criteria of the water deprivation test were based on data from only 36 patients (28) and, unsurprisingly, the overall diagnostic accuracy of this test is very poor (diagnostic accuracy 70%) (42). Moreover, to reliably interpret AVP action or urine concentration capacity, a hypernatremic state has to be reached. This is often not the case during the water deprivation test (42), as exemplified by our case. With the hypertonic saline test, a hypernatremic state is ensured and the measurement of stimulated copeptin values increases the diagnostic accuracy of the test to 97% (43).

Primary polydipsia has traditionally been linked to psychiatric disease, such as schizophrenia spectrum disorder (125), but is increasingly reported in healthy people without psychiatric comorbidities (40, 126, 127), as it was the case for our patient. In clinical practice, cognitive behavioral therapy is used to treat primary polydipsia and was successful in our patient. However, guidelines for the management of this syndrome are lacking and medical treatment options are scarce (128). We have recently shown that a 3-week treatment with the glucacon-like peptide-1 receptor agonist dulaglutide reduces thirst, daily fluid intake, and 24-hour urine output in primary polydipsia (126). This proof of concept study provides a novel therapeutic approach and the GLP-1 receptor agonist may in the future become an interesting treatment option for these patients.

## Conclusion

In conclusion, copeptin is secreted in equimolar amounts to AVP and reliably mirrors AVP concentrations in the circulation. Its measurement has several advantages over AVP since it is stable ex vivo, can easily be measured, and results are available within a few hours. The 2 assays with sufficient technical description and clinical data justifying their routine clinical use are the sandwich LIA (3) and its automated immunofluorescent successor on the KRYPTOR platform.

The main and best-validated indication for copeptin measurement is in the differential diagnosis of polyuria–polydipsia syndrome. Here, baseline levels >21 pmol/L identify patients with nephrogenic diabetes insipidus and osmotically stimulated levels with hypertonic saline (cut-off 4.9 pmol/L) or nonosmotically stimulated levels with arginine (cut-off 3.8 pmol/L) differentiate between central diabetes insipidus and primary polydipsia with a high diagnostic accuracy.

In hyponatremia, the diagnostic utility of copeptin is of minor relevance and may be limited to fast and reliable identification of patients with underlying primary polydipsia. No differentiation between SIAD and other etiologies of hyponatremia seems possible, nor can copeptin reliably differentiate between cancer-related SIAD and other etiologies.

In hypernatremia, copeptin measurements below 4.4 pmol/L are diagnostic for central diabetes insipidus and allow identifying patients in need of desmopressin treatment.

Of note, in clinical settings where copeptin assays are unavailable or not available on the same day, close clinical supervision and judgment of routinely measured plasma and urine parameters are still the mainstay of differential diagnosis of disturbances in water homeostasis. Copeptin has also emerged as an unspecific marker of stress, predicting outcome in patients with sepsis, ischemic stroke, myocardial infarction, or pneumonia, among others. In patients with type 1 and type 2 diabetes, copeptin seems to be a predictive marker for the development of diabetic kidney disease. In addition, in ADPKD, it is suggested that copeptin predicts disease progression and treatment response to vaptans. Whether measurement of copeptin in these diseases has real clinical implications remains to be shown in future studies.

## Data Availability

Data sharing is not applicable to this article as no datasets were generated or analyzed during the current study.

## Abbreviations

ADPKD: autosomal dominant polycystic kidney disease
AVP: arginine vasopressin
ELISA: enzyme-linked immunosorbent assay
IQR: interquartile range
LIA: immunoluminometric assay
SIAD: syndrome of inappropriate antidiuresis
